# Pb(II)-inducible proviolacein biosynthesis enables a dual-color biosensor toward environmental lead

**DOI:** 10.3389/fmicb.2023.1218933

**Published:** 2023-07-27

**Authors:** De-long Zhu, Yan Guo, Bing-chan Ma, Yong-qin Lin, Hai-jun Wang, Chao-xian Gao, Ming-qi Liu, Nai-xing Zhang, Hao Luo, Chang-ye Hui

**Affiliations:** ^1^School of Public Health, Guangdong Medical University, Dongguan, China; ^2^Shenzhen Prevention and Treatment Center for Occupational Diseases, Shenzhen, China; ^3^School of Public Health, Tongji Medical College, Huazhong University of Science and Technology, Wuhan, China

**Keywords:** whole-cell biosensors, proviolacein biosynthesis, lead pollution, bioavailability, ecotoxicity

## Abstract

With the rapid development of synthetic biology, various whole-cell biosensors have been designed as valuable biological devices for the selective and sensitive detection of toxic heavy metals in environmental water. However, most proposed biosensors are based on fluorescent and bioluminescent signals invisible to the naked eye. The development of visible pigment-based biosensors can address this issue. The *pbr* operon from *Klebsiella pneumoniae* is selectively induced by bioavailable Pb(II). In the present study, the proviolacein biosynthetic gene cluster was transcriptionally fused to the *pbr* Pb(II) responsive element and introduced into *Escherichia coli*. The resultant biosensor responded to Pb(II) in a time- and dose-dependent manner. After a 5-h incubation with Pb(II), the brown pigment was produced, which could be extracted into n-butanol. Extra hydrogen peroxide treatment during n-butanol extract resulted in the generation of a stable green pigment. An increased brown signal was observed upon exposure to lead concentrations above 2.93 nM, and a linear regression was fitted from 2.93 to 3,000 nM. Extra oxidation significantly decreased the difference between parallel groups. The green signal responded to as low as 0.183 nM Pb(II), and a non-linear regression was fitted in a wide concentration range from 0.183 to 3,000 nM. The specific response toward Pb(II) was not interfered with by various metals except for Cd(II) and Hg(II). The PV-based biosensor was validated in monitoring bioaccessible Pb(II) spiked into environmental water. The complex matrices did not influence the regression relationship between spiked Pb(II) and the dual-color signals. Direct reading with the naked eye and colorimetric quantification enable the PV-based biosensor to be a dual-color and low-cost bioindicator for pollutant heavy metal.

## Introduction

Heavy metal pollution has become a global environmental problem that seriously endangers human health and ecological safety (Adimalla, 2020; Zhu et al., 2022). Some heavy metals, including lead (Pb), mercury (Hg), arsenic (As), chromium (Cr), and cadmium (Cd), can cause significant damage to multiple organs such as the liver, kidneys, and brain by disrupting the body’s metabolic functions, and lead to different types of cancer, neurological disorders, and other endocrine abnormalities (Bridges and Zalups, 2017; Klotz and Goen, 2017; Rehman et al., 2018). As one of the critical heavy metal pollutants, Pb can quietly threaten human life through various hidden pathways (Mulhern et al., 2022). Due to its high toxicity and non-biodegradability (Yuvaraja et al., 2019), Pb can accumulate in living organisms through the food chain and threaten their bioactivities (Naikoo et al., 2019). Pb is toxic to almost all human organs (Charkiewicz and Backstrand, 2020; Hemmaphan and Bordeerat, 2022). Thus, the biological exposure limit of blood lead is constantly being lowered from 60 μg/dL in 1960 to 3.5 μg/dL in 2016 (Rocha and Trujillo, 2019).

Implementation of rational control policies and continuous environmental monitoring are prerequisites for avoiding increased Pb pollution (Pohl et al., 2017). Accurate detection of bioavailable Pb in soil and water is necessary to predict its ecological risk (Roux and Marra, 2007; Kumar et al., 2020). From available studies, most methods for detecting Pb rely on expensive instruments that are not portable and limited to specific analytical systems (Klotz and Goen, 2017; Tudosie et al., 2021; Manousi et al., 2022). Although the mainstream instrumental methods are sensitive and accurate, the analytical results mainly reflect the total amount of elemental Pb. The bioavailable Pb that is genuinely hazardous to organisms was not exactly reflected using traditional instruments, including atomic absorption spectroscopy and inductively coupled plasma-mass spectrometry. The specification analysis of toxic Pb largely depends on complex pre-treatment (Manousi et al., 2022). As an alternative to instrumental assays, the novel microbial cell-based approach showed potential in the practical determination of bioavailable heavy metals (Cui et al., 2018; Hui et al., 2020). These biological methods also have the advantage of solid operability, low cost, and ease of use (Kannappan and Ramisetty, 2022). Despite the lack of rigorous selectivity and background noise, microbial cell-based biosensors can be continuously improved by evolving genetic techniques to meet on-site testing requirements (Wang and Buck, 2012).

Bioaccessible Pb(II) is critical to the transcription initiation of Pb resistance genes, mediated by the metalloregulator PbrR (Borremans et al., 2001). The Pb(II)-resistant *pbr* operon, a member of the *mer*-like operons, was commonly engineered to develop several microbial cell-based biosensors toward Pb (Fang and Zhang, 2022). With the rapid advances in synthetic biology, it is now possible to artificially reconstruct heavy metal-resistant operons, including optimization of the sensory modules (Levin-Karp et al., 2013; Jia et al., 2020) and development of novel reporter modules (Jiang et al., 2008). When bioavailable metals enter the biosensing cell, the transcription and translation of downstream reporter genes will be induced, and the resultant biosensing signals can be emitted. Various biosensing reporters are employed to develop genetic devices responsive to heavy metal pollutants. Versatile engineered microbial cells became bioluminescent biosensors (Viviani et al., 2022), fluorescent biosensors (Guo et al., 2021b; Hui et al., 2021b), and colorimetric biosensors (Hui et al., 2021a) toward toxic metals.

Bioluminescent and fluorescent reporters as primary actuators depend highly on expensive instruments to determine biosensing signals (Hui et al., 2021b; Viviani et al., 2022). These instruments, such as photometers, multi-wavelength microplate readers, and fluorescence spectrometers, are not conducive to rapidly detecting Pb contamination in the field. Recently, several natural pigments as easy-to-read biosensing reporters were successfully designed to develop whole-cell biosensors for the detection of environmental Hg(II) (Hui et al., 2021a), Cd(II) (Hui et al., 2022a) and Pb(II) (Hui et al., 2022b). These novel pigment-based biosensors have great potential in developing rapid colorimetric methods for *in situ* detection of contaminant metals, with low-cost, mini-equipment, and high-throughput advantages.

Pigments with striking colors could be used to develop visual bacterial biosensors toward pollutants. Several studies have clarified that the branched violacein biosynthetic pathway can produce four-color metabolites, including violacein (V), deoxyviolacein (DV), proviolacein (PV), and prodeoxyviolacein (PDV) (Yang et al., 2021). Differential colored signals contribute to the design of multiple functional biosensors. Whole-cell biosensors based on navy V (Hui et al., 2020) and purple DV (Hui et al., 2022b) showing differential responsive properties have been successfully developed. The PV-based colored signal responsive to Pb(II) was previously obtained by employing a *vioABDE* gene cluster as a reporter module fused downstream of the Pb(II) sensory element. Due to the high reducibility of the chromogenic compounds, the color rendering of PV is unstable. Thus its feasibility as a biosensing reporter has yet to be verified in previous studies. In the current study, The PV biosynthesis was demonstrated to be selectively activated by soluble Pb(II) in a time- and dose–response manner. Due to the extreme reducibility of PV, green and brown PV derivatives were generated under oxidizing and non-oxidizing conditions, and the dual-color signals could be quantified by visible light colorimetry. A high-throughput colorimetric method based on a 96-well plate was developed to detect environmental Pb pollutants. It has the potential to become a complementary tool to instrumental means.

## Materials and methods

### Biosensing construct, bacterial strains, and reagents

The biosensing construct pPb-vioABDE with a *vioABDE* gene cluster located downstream of the Pb(II) sensory element was previously constructed (Hui et al., 2022b). In brief, the Pb(II) sensing element contains a reverse transcriptional *pbrR* gene and divergent *pbr* promoter originating from *Klebsiella. pneumoniae* CG43 (NCBI Accession AY378100) was artificially synthesized (Sangon Biotech, Shanghai, China). The proviolacein biosynthetic genes originating from *Chromobacterium violaceum* were designed according to the preference codon of *E. coli* and synthesized (Sangon Biotech). An overlap PCR assembled two genetic modules and inserted them into the *Bgl*II and *Sac*I sites of pET-21a to generate the vector pPb-vioAEDE. *E. coli* TOP10 was used as the bacterial host and transformed with pPb-vioABDE to assemble bacterial biosensor TOP10/pPb-vioABDE. Chemically inert violacein and deoxyviolacein have been validated as becoming potential reporters in our previous studies (Hui et al., 2020, 2022b). The biosensing performance of unstable proviolacein was investigated in the current study. More colored pigment choices are beneficial for developing versatile biosensors for detecting toxic metals of different concentrations or even types. Engineered strains were grown in Luria-Bertani (LB) broth (10 g/L tryptone, 5 g/L yeast extract, 10 g/L NaCl, and 50 μg/mL ampicillin) in a 15 mL bio-reaction tube with a vent cap (Jet Bio-Filtration, Guangzhou, China) at 37°C with shaking at 250 rpm under aerobic conditions. Inorganic metal compounds, including HgCl_2_, CaCl_2_, MgCl_2_, FeSO_4_, MnSO_4_, NiSO_4_, CuSO_4_, ZnSO_4_, CdCl_2_, and Pb(NO_3_)_2_ were analytical grade reagents and obtained from Sigma Aldrich (St. Louis, MO, United States). Various metal stock solutions were freshly prepared using purified water at 1 mM. Other chemical reagents were purchased from Sangon Biotech (Shanghai, China).

### Chromogenic stability of two colored PV derivatives

Overnight cultures of TOP10/pPb-vioABDE were inoculated into 10 mL fresh LB medium at a 1:100 dilution and induced with 1 μM Pb(II) at 37°C for 5 h. Intracellular accumulated PV aggregates were extracted with n-butanol. After vortex extraction using 4 mL n-butanol, the n-butanol phase was separated after centrifugation and divided into the average non-oxidation and oxidation treatment groups. The difference was that 30% H_2_O_2_ in a ratio of 1:9 was supplemented into the oxidation group during n-butanol extraction. Both n-butanol extracts were placed at 25°C and sampled at 1-h intervals. The visible absorption spectra of the samples were scanned in a microplate reader (BioTek Epoch, USA) ranging from 400 to 700 nm at 1-nm intervals.

### The time-dose–response pattern of PV-based biosensors toward Pb(II)

Overnight cultures of TOP10/pPb-vioABDE were inoculated into 2 mL LB medium at a ratio of 1:100 in 15 mL tubes, then exposed to Pb(II) at final concentrations of 0, 0.15, and 1.5 μM. The induced cultures were incubated at 37°C with shaking at 250 rpm for 8 h and sampled at 1-h intervals. All samples were divided into oxidation and non-oxidation treatment groups on average and stored at 4°C until detection. Aliquots of 100 μL culture were first determined at 600 nm for the bacterial density (OD_600_). The residual 900 μL culture was directly extracted with 360 μL n-butanol in non-oxidation treatment groups; the residual 900 μL culture was extracted with 360 μL n-butanol mixed with 100 μL 30% H_2_O_2_ in oxidation treatment groups. After being vigorously vortexed for 2 min, the upper n-butanol phase was separated by centrifugation at 12000 × g for 1 min and placed at room temperature to ensure the sufficient derivation of PV. Then, aliquots of 100 μL organic phase were transferred into a 96-well microplate and read at 652 nm to determine the dual-color signals using a microplate reader.

### Dose–response curves for Pb(II)-induced PV-based biosensors

Overnight cultures of TOP10/pPb-vioABDE were diluted at 1:100 in fresh LB medium. A 2-fold dilution method (Hui et al., 2021c) was used to obtain 12,000, 6,000, 3,000, 1,500, 750, 375, 187.5, 93.8, 46.9, 23.4, 11.7, 5.86, 2.93, 1.46, 0.732, 0.366, 0.183, 0.0915 and 0 nM Pb(II) exposure group. After incubation at 37°C for 5 h, an aliquot containing 100 μL cultures was measured for bacterial density at 600 nm. Intracellular PV aggregates were extracted with n-butanol after oxidation and non-oxidation treatment, as described above. An aliquot containing 100 μL organic phase was measured at 652 nm to determine the dual-color signals.

### Response selectivity of PV-based biosensors

To investigate the response selectivity, the overnight cultures of TOP10/pPb-vioABDE were diluted at 1:100 in fresh LB medium and exposed to 1.5 μM various metal ions, including Pb(II), Cr(III), Hg(II), Zn(II), Mg(II), Ni(II), Mn(II), Ca(II), Fe(II), Cd(II), and Cu(II). To assess the influence of various metal ions on the response of TOP10/pPb-vioABDE toward Pb(II), target Pb(II) was mixed with various interference metal ions at 1.5 μM and exposed to biosensor cells. After incubation at 37°C for 5 h, the bacterial cell density and dual-color signals were measured at 600 nm and 652 nm, respectively.

### Detection of bioavailable Pb(II) in environmental samples

Lead salt was spiked into four environmental water samples: pure water, tap water, lake water, and soil extracts. The response of PV-based biosensors toward bioaccessible Pb(II) in the environmental matrices was investigated. Environmental surface water and surface (0–20 cm) lateritic soil samples were collected from a local park (Luohu District, Shenzhen, China). Air-dried soil samples (10 g) were fully suspended using 200 mL of pure water and vortexed for 1 h. After being placed at 26°C for 5 h, the supernatant was filtered through a 0.22 μM filter. The LB medium was freshly prepared by mixing 90% v/v filtered purified water, tap water from our laboratory, ambient surface water, and soil extract with 10% v/v 10 × LB broths. Overnight cultures of TOP10/pPb-vioABDE were diluted at 1:100 in four media. A double dilution method was used to obtain 3,000, 1,500, 750, 375, 187.5, 93.8, 46.9, 23.4, 11.7, 5.86, 2.93, and 0 nM Pb(II) exposure groups for non-oxidation treatment, and 6,000, 3,000, 1,500, 750, 375, 187.5, 93.8, 46.9, 23.4, 11.7, 5.86, 2.93, 1.46, 0.732, 0.366, 0.183, 0.0915 and 0 nM Pb(II) exposure groups for oxidation treatment. After incubation at 37°C for 5 h, bacterial density and dual-color signals were determined at 600 nm and 652 nm, respectively.

## Results

### Pb(II)-initiated proviolacein synthesis resulting in the accumulation of chromogenic substances

**Figure 1:**
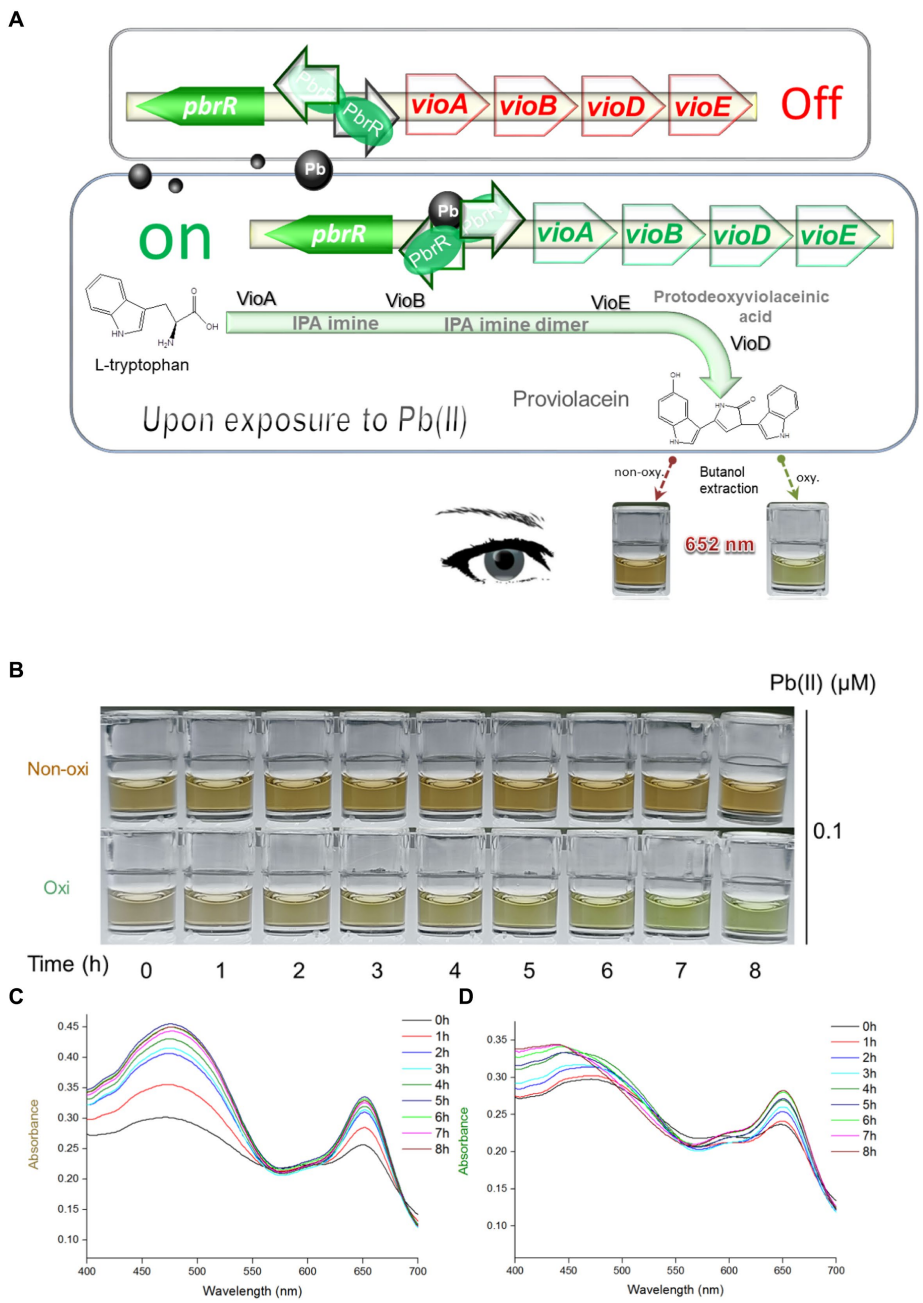
Pb(II) triggers the biosynthesis of proviolacein, which can be converted into two colored substances under non-oxidizing and oxidizing conditions. **(A)** Design of PV-based whole-cell biosensor toward Pb(II). The PV biosynthetic module is artificially synthesized and genetically fused downstream of the Pb(II) sensory module. A tetracistronic unit is transcribed upon exposure to intracellular bioavailable Pb(II). PV biosynthesis is based on the branched violacein biosynthetic pathway catalyzed by VioA, VioB, VioE, and VioD. After n-butanol extraction accompanied by oxidation and non-oxidation treatments, PV in the organic phase was converted into two colored substances. PbrR, Pb(II) responsive metalloregulator; IPA, indole-3-pyruvate acid imine; oxy, oxidizing condition; non-oxy, non-oxidizing condition. **(B)** After non-oxidation and oxidation treatment, the n-butanol phase containing PV was placed at 25°C for 8 h. Representative photos from three independent experiments are shown. Visible absorption spectra of PV derivatives after non-oxidation **(C)** and oxidation **(D)** treatment were scanned at 1-h intervals. The wavelength range is from 400 to 700 nm in 1 nm intervals. Representative results from three independent assays are shown.

Pb(II) sensory element originating from the natural lead-resistant bacterium *Klebsiella. pneumoniae* CG43 was transcriptionally fused with the PV biosynthesis genes cluster to generate a biosensing construct of the plasmid pPb-vioABDE. The molecular mechanism of Pb(II) inducible PV biosynthesis is shown in Figure 1A. Bacterial cells usually acquire metal ions through the ATP-binding cassette transporters, which lack transport selectivity and transport various metal ions, including Pb(II) inside a bacterial cell (Locher, 2016). Intracellular Pb(II) can transform dimeric PbrR from a repressor into an activator. Then the *vioABDE* gene cluster is transcribed and translated in a Pb(II) concentration-dependent manner, resulting in the activated metabolic flux toward proviolacein forms in the cytoplasm.

As an intermediate of the branched violacein biosynthetic pathway, the unstable PV in the organic phase was rapidly converted into a grayish-brown substance upon exposure to air (Figure 1B), and brown gradually darkened under autoxidation conditions (non-oxidation treatment group). However, the PV in the organic phase was gradually converted into a green substance within 8 h under the oxidation of intense oxidant hydrogen peroxide (oxidation treatment group). The visible absorption spectra in the 400–700 nm wavelength showed that the absorption values of two colored derivatives increased with the prolonged incubation time. The rising trend of the non-oxidation group (Figure 1C) was significantly more evident than that of the oxidation group (Figure 1D). Importantly, the absorbance of two colored PV derivatives became invariant after 6-h incubation. Both colored PV derivatives showed two prominent absorption peaks. A 400 to 550 nm peak was flat, but another sharp peak was located at about 652 nm. According to the above results, unchanging absorbance of 652 nm could be determined after the n-butanol phases containing PV derivatives were placed for 6 h. In the subsequent experiments, stable brown and green signals were determined after 6 h following n-butanol extraction under non-oxidizing and oxidizing conditions.

### PV-based biosensors responsive to Pb(II) in a time-dependent manner

**Figure 2:**
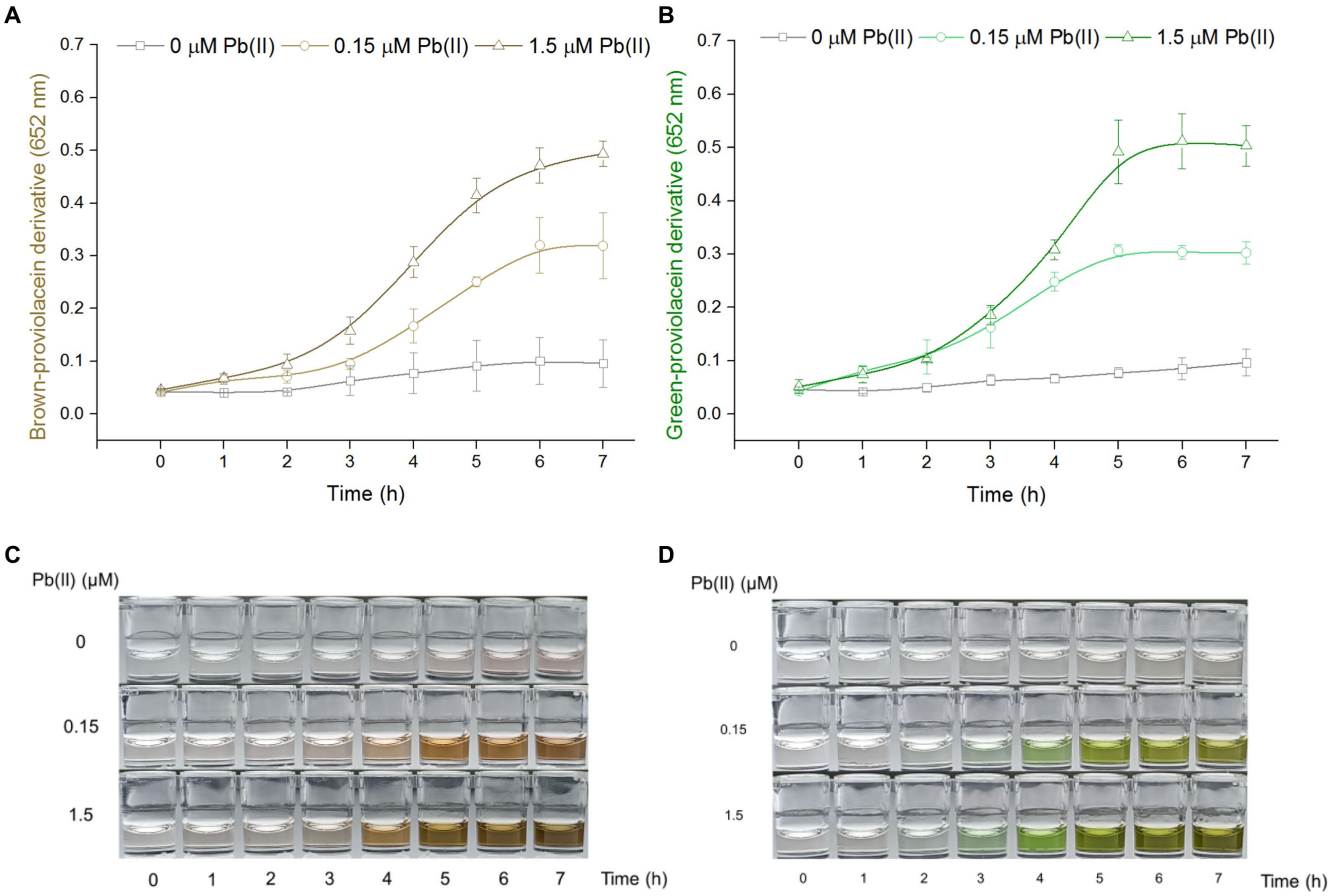
The time-dose–response relationship of PV-based biosensors exposed to Pb(II). Cultures of TOP10/pPb-vioABDE were induced with three concentrations of Pb(II) at 37°C and sampled at 1-h intervals. The n-butanol extracts were separated and transferred into a 96-well plate. The time-dose–response curves using A_652_ of 100 μL n-butanol phase after non-oxidation **(A)** and oxidation **(B)** treatment were made. The result was the mean ± standard deviation of three independent determinations. Representative photos of two colored n-butanol phases from non-oxidation treatment **(C)** and oxidation treatment **(D)** groups are shown.

Engineered TOP10/pPb-vioABDE was exposed to Pb(II) at concentrations of 0, 0.15, and 1.5 μM, bacterial density increased within 7 h, and the A_652_ of the n-butanol phases containing PV derivatives showed an apparent time-dose–response relationship in both non-oxidation treatment groups (Figure 2A), and oxidation treatment groups (Figure 2B). The induction coefficient (A_652_ of Pb(II) exposure group / A_652_ of no Pb(II) exposure group) in the non-oxidation treatment group and oxidation treatment group is shown in Supplementary Tables S1, S2, respectively. The induction coefficient continuously increased with 7 h Pb(II) induction in a non-oxidation group (Supplementary Table S1). However, the oxidation group’s induction coefficient reached the maximum at 5 h Pb(II) induction (Supplementary Table S2). The green signal was also stable after 5 h Pb(II) induction (Figure 2B). Interestingly, the standard deviations of the non-oxidation treatment group were more extensive than that of the oxidation treatment group upon exposure to 0 and 0.15 μM Pb(II). The n-butanol phase containing PV derivatives changed from colorless to light brown. The brown continued to deepen until 6 h induction in the non-oxidation treatment group (Figure 2C). By comparison, the n-butanol phase containing PV derivatives darkened to a stable brown-green after 5 h Pb(II) induction in the oxidation treatment group (Figure 2D). Finally, five h Pb(II) induction was chosen before the determination of dual-color signals in the subsequent experiments.

### PV-based biosensors responsive to Pb(II) in a dose-dependent manner

**Figure 3:**
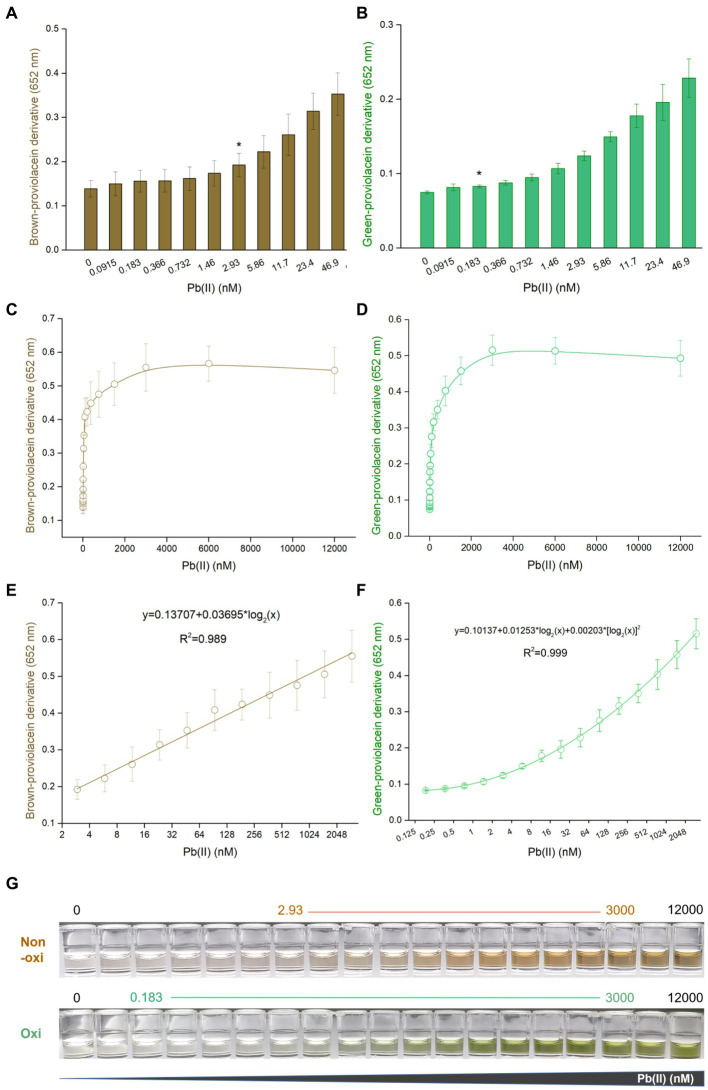
Dose–response performance of PV-based biosensor toward Pb(II) under laboratory conditions. Comparison of response sensitivity of TOP10/pPb-vioABDE toward Pb(II) between the non-oxidation treatment group **(A)** and oxidation treatment group **(B)**. The asterisk indicates LOD (the absorbance at 2.93 or 0.183 nM Pb(II) exposure group was checked against the background by t-Student analysis, and the difference was statistically significant (*P* < 0.05)) representing the lowest Pb(II) concentration inducing significantly increased pigment biosynthesis. Dose–response curves are based on the brown signal, captured after non-oxidation treatment **(C)**, and the green signal, captured after oxidation treatment **(D)**. **(E)** Linear regression analysis of the brown signal and Pb(II) relationship ranging from 2.93 to 3,000 nM (*R*^2^ = 0.989). **(F)** Non-linear regression analysis of the relationship between the green signal and Pb(II) ranging from 0.183 to 3,000 nM (*R*^2^ = 0.999). The *x*-axis shows the Pb(II) concentration on the log_2_ scale. The result was the mean ± standard deviation of three independent assays. **(G)** Representative photos of two colored n-butanol phases are shown, and the Pb(II) concentration ranges of regression analysis are marked.

Engineered TOP10/pPb-vioABDE was exposed to increased concentrations of Pb(II). Pb(II) concentrations below 12,000 nM did not adversely affect bacterial growth (Supplementary Figure S2). The brown signal was unstable in the non-oxidation treatment group with a significant relative standard deviation. Thus, a significantly increased brown signal was observed upon exposure to 2.93 nM Pb(II) (Figure 3A). However, the green signal was stable with a slight relative standard deviation, so the limit of detection (LOD) was decreased to 0.183 nM after oxidation treatment (Figure 3B). The biosynthesis of PV was Pb(II) dose-dependently induced. The dose–response curves were similar between the non-oxidation (Figure 3C) and oxidation (Figure 3D) treatment groups. The brown signal was not increased above 1,024 nM Pb(II) induction, and the green signal was not above 2048 nM Pb(II) induction. The brown signal and Pb(II) exposure concentration tended to be fitted to a linear regression ranging from 2.93 to 3,000 nM (Figure 3E). After oxidation treatment, the green signal and Pb(II) exposure concentration tended to be fitted to a non-linear regression with a wide concentration range from 0.183 to 3,000 nM (Figure 3F). As shown in Figure 3G, the background (no Pb exposure) of the oxidation treatment group was almost colorless to the naked eye, but slight brown was observed in the background of non-oxidation treatment group. Both colors were visually deepened with the increased Pb(II) exposure.

### PV-based biosensors selectively respond to toxic Pb(II)

**Figure 4:**
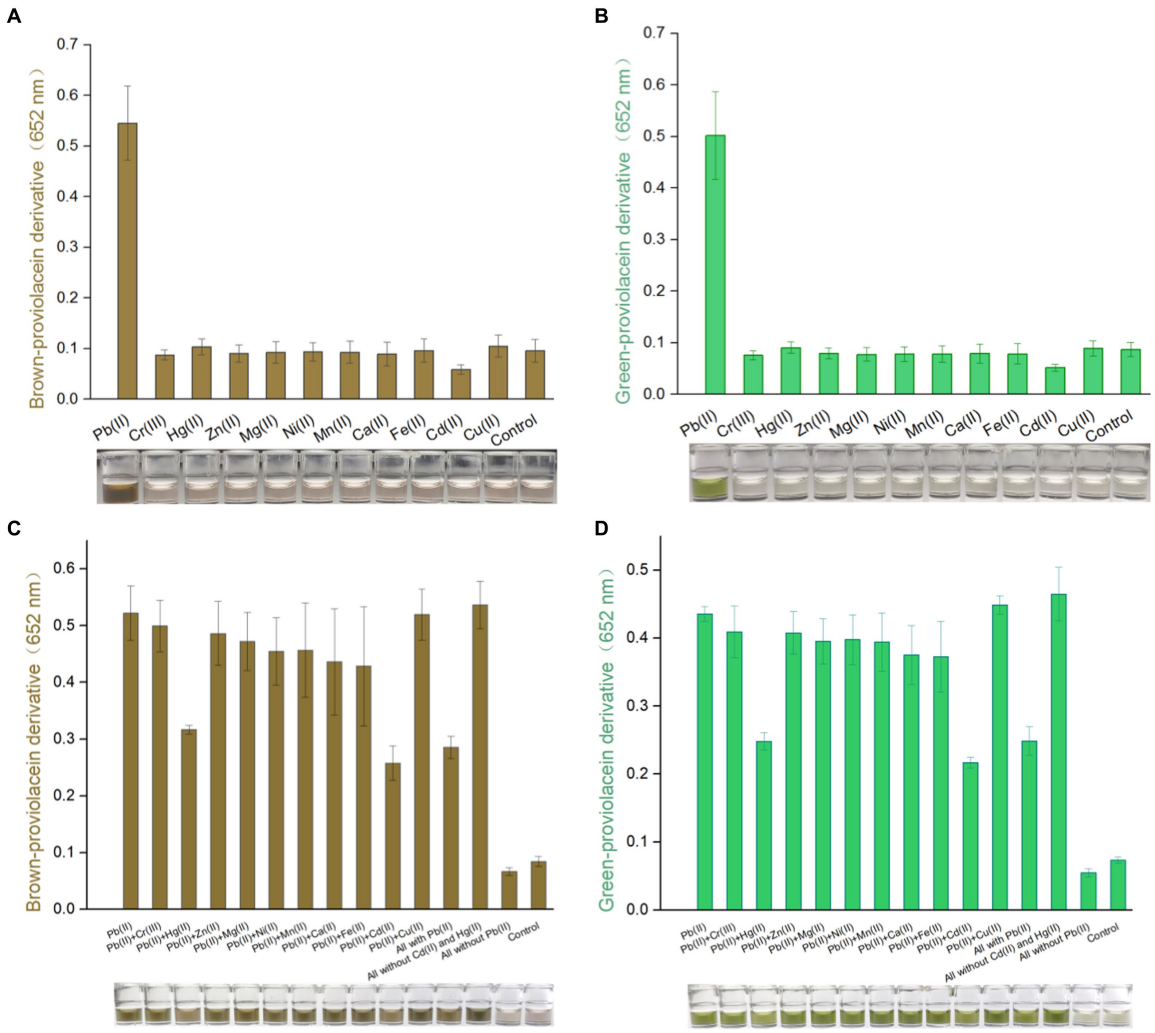
Differential responses of PV-based biosensors in response to various metal ions. Various metal ions induced TOP10/pPb-vioABDE at a concentration of 1.5 μM. The brown signal **(A)** and the green signal **(B)** were read at 652 nm following non-oxidation and oxidation treatment. Various metal mixtures induced TOP10/pPb-vioABDE at a concentration of 1.5 μM. The brown signal **(C)** and the green signal **(D)** were determined following non-oxidation and oxidation treatment. The control group was set without metal exposure. The result was the mean ± standard deviation of three independent assays. Representative photos of the n-butanol phase are shown below the bar chart.

Engineered TOP10/pPb-vioABDE was exposed to Pb(II) and ten other metal ions at 1.5 μM. Supplement of various metal ions at 1.5 μM exerted no apparent cytotoxicity on bacterial cells (Supplementary Figures S3A,B). PV-based biosensors selectively responded to Pb(II). Notably, the non-oxidation treatment (Figure 4A) and oxidation treatment (Figure 4B) did not influence the output of biosensors’ selectivity. Upon exposure to Cd(II), a decreased background response compared to other non-target metal ions was observed. The effect of coexisting metals exerted slight cytotoxicity on biosensor cells (Supplementary Figures S3C,D). Various metal ions’ interaction with an accumulation of colorant was further investigated. In both non-oxidation (Figure 4C) and oxidation (Figure 4D) treatment groups, coexisting Hg(II) and Cd(II) impaired the response of TOP10/pPb-vioABDE toward Pb(II). All used metals together weakened the biosensing response to Pb(II), possibly due to the existence of Hg(II) and Cd(II). The production of PV responsive to Pb(II) was restored exactly when Hg(II) and Cd(II) were eliminated from all used metals. Notably, the brown and green color in n-butanol phases visible to the naked eye were still observed in all exposure groups containing Pb(II).

**Figure 5:**
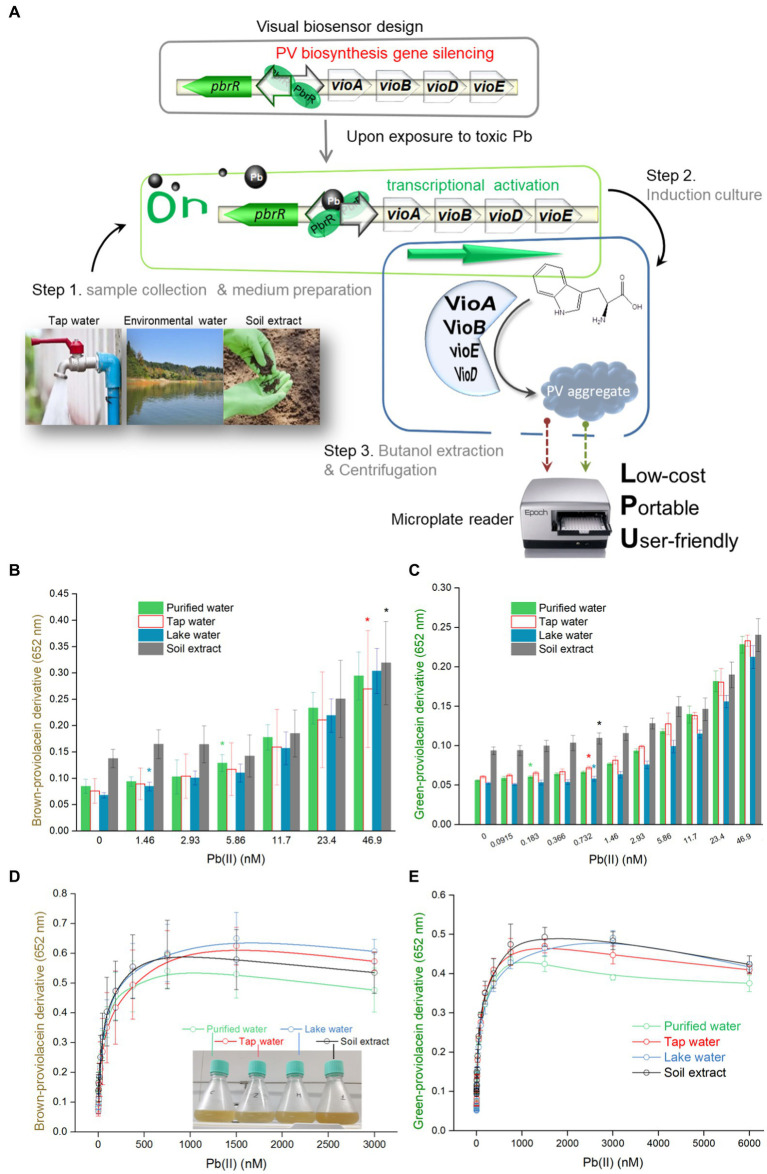

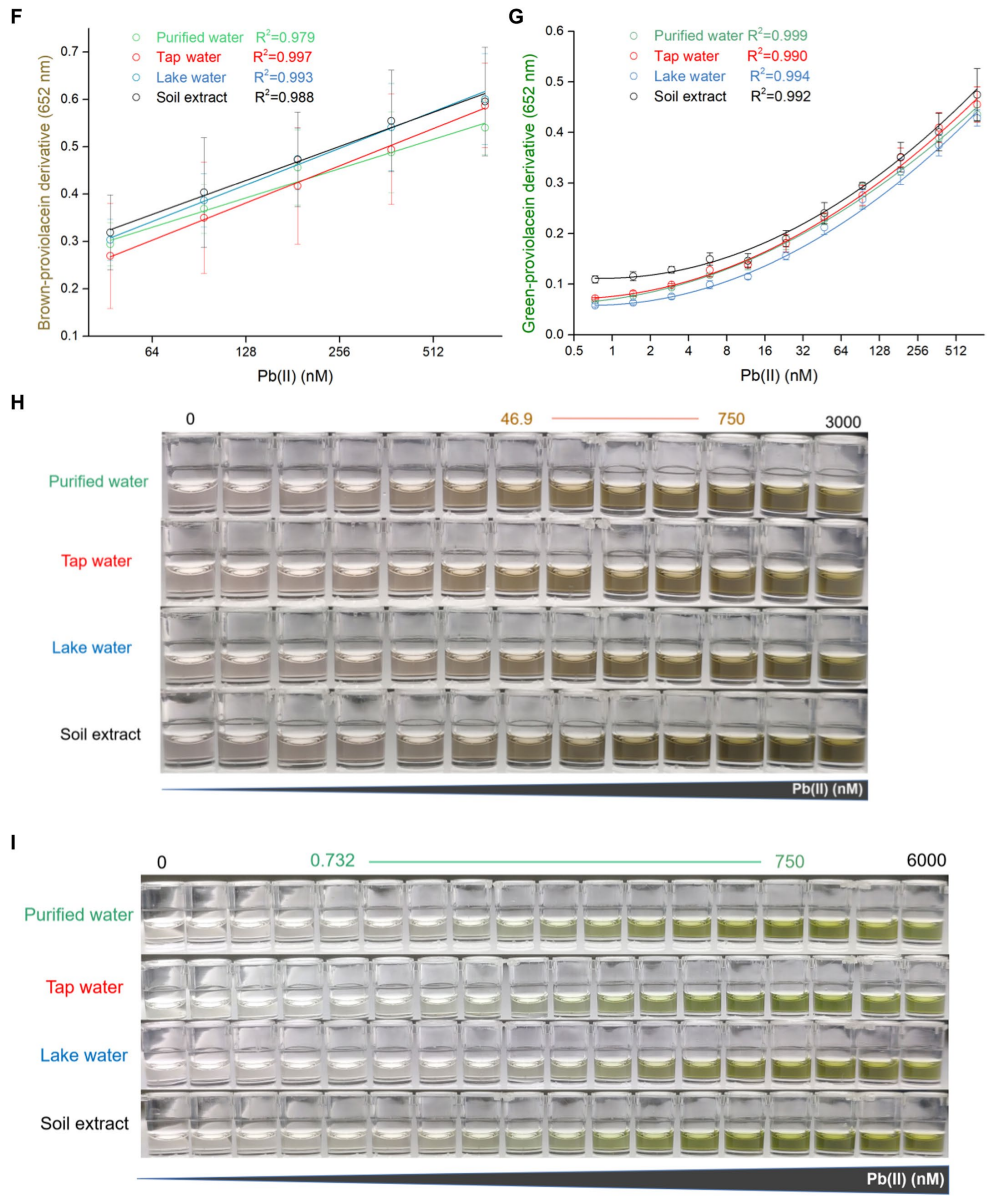
Dose–response performance of PV-based biosensor in detecting Pb(II) in environmental samples. **(A)** A detailed protocol for environmental Pb(II) bioindication using PV-based biosensors. Engineered TOP10/pPb-vioABDE was cultured in LB medium prepared using four different water samples, including purified water, tap water, lake water, and soil extract. Comparison of response sensitivity of TOP10/pPb-vioABDE toward Pb(II) between the non-oxidation treatment group **(B)** and oxidation treatment group **(C)** in the four culture substrates. The different colored asterisk indicates LOD (the absorbance was checked against the background by t-Student analysis, and the difference was statistically significant (*P* < 0.05)), representing the lowest Pb(II) concentration inducing significantly increased pigment biosynthesis in four cultures. The dose–response curves based on the resultant brown signal **(D)** and the resultant green signal **(E)** were drawn after non-oxidation and oxidation treatment. **(F)**Linear regression analysis of the relationship between the brown signal and Pb(II) concentrations ranging from 46.9 to 750 nM. **(G)** Non-linear regression analysis of the relationship between the green signal and Pb(II) concentrations ranging from 0.732 to 750 nM. The inset shows the R^2^ values of the regression equations. The *x*-axis shows the Pb(II) concentration on the log_2_ scale. The result was the mean ± standard deviation of three independent assays. Representative photos of the n-butanol phases containing PV derivatives after non-oxidation **(H)** and oxidation **(I)** treatment are shown, and the Pb(II) concentration ranges of regression analysis are marked.

### Bioavailable Pb(II) in environmental water matrices is detected using PV-based biosensors

In order to validate the capability of engineered biosensors in monitoring bioavailable Pb(II) in different matrices, environmental water samples artificially contaminated with Pb(II) were mixed into the culture systems. The overall procedure for the Pb(II) field testing is shown in Figure 5A. Bacterial density was stable with 0–6,000 nM Pb(II) exposure (Supplementary Figure S4). Considering soluble Pb(II) cytotoxicity, bacterial viability was calculated by spreading serial ten-fold dilution cultures on the LB plates containing 50 μg/mL ampicillin. Compared with the control group (no Pb exposure), the number of colony-forming units (CFU) slightly decreased in high concentrations of Pb(II) induced bacterial culture (Supplementary Figure S5). The concentration range of Pb(II) used in the study has no significant lethal effect on bacteria.

Brown signal fluctuations caused by environmental matrix effects (Figure 5B) were significantly higher than green signal fluctuations (Figure 5C). Compared with the green signal, an significantly increased brown signal was observed with higher concentrations of Pb(II) exposure. The overall dose–response curves were highly similar at low concentrations of Pb(II) exposure (Figures 5D,E). Similar to the regression analysis under experimental conditions, a linear regression relationship was found in the non-oxidation treatment group with Pb(II) concentration ranging from 46.9 to 750 nM (Figure 5F). A non-linear regression was demonstrated in the oxidation treatment group with Pb(II) concentration ranging from 0.732 to 750 nM (Figure 5G). Furthermore, the relative standard deviations were significantly decreased in the oxidation treatment group (Supplementary Tables S3, S4). A high background was found in the non-oxidation treatment group (Figure 5H). In contrast, a more pronounced gradient of green color was observed in the oxidation group (Figure 5I).

## Discussion

Microorganisms are inexpensive weapons for defending against heavy metal pollution, as they can adapt to high concentrations of heavy metal exposure through metal ions sequestration, transport, and export (Dave et al., 2020). The primary heavy metal pollutants in environmental water include Pb(II), Hg(II), Cd(II), As(III), and Cr(III) (Kiran Marella et al., 2020). Recently, biological assays have been extensively investigated to detect residual heavy metals in surface water or soil using genetically modified bacteria. Compared with non-cellular devices such as protein-based biosensors, DNA-based biosensors, and electrochemical sensors, whole-cell biosensors can potentially monitor cytotoxic, bioavailable, and bioaccessible metals in a low-cost, mini-equipment, and high-throughput manner. Various whole-cell biosensors have been successfully developed with fluorescent (Hui et al., 2021b; Kolosova et al., 2022), and colorimetric (Hui et al., 2021a) signal outputs.

Recent studies showed that natural pigment biosynthesis based on metabolic engineering made designed bacteria become colorimetric biosensors toward pollutants (Watstein and Styczynski, 2017; Hui et al., 2020, 2021a, 2022c; Guo et al., 2021a). Redesign of the metabolic pathway facilitated the biosynthesis of interested colorants (Hui et al., 2022b). The colored PV could be selectively accumulated by interrupting the branched violacein biosynthetic pathway (Tong et al., 2021; Hui et al., 2022b). The PV biosynthetic gene cluster was employed as a pigment-based reporter and transcriptionally fused to the well-characterized Pb(II) sensing element. The resultant engineered bacterium was expected to become a PV-based Pb(II) biosensor. As an intermediate of the violacein biosynthetic pathway, PV is chemically active (Zhao et al., 2019). Its extreme reducibility was first demonstrated in the present study. Bimodal PV derivatives with two peaks at about 480 nm and 652 nm were gradually generated, and stable dual-color signals could be captured at about 6 h after extraction into butanol (Figures 1C,D). Our findings shew that natural oxidation led to the accumulation of brown-colored PV derivatives in the n-butanol phase. Unexpectedly, more stable green-colored PV derivatives were predominantly generated due to the rapid and intense oxidation mediated by hydrogen peroxide. Although background signal noise is difficult to eliminate in whole-cell biosensors employing natural metalloregulators as sensory elements (Bereza-Malcolm et al., 2016; Hui et al., 2021a), the results showed that the intense oxidant treatments facilitated stabling and decreasing the background in the control group and reducing signal fluctuations in parallel groups (Figure 2).

Due to the stable green signal, the PV-based biosensor had a low LOD of 0.183 nM after an extra oxidation treatment (Figure 3B). However, the LOD was increased to 2.93 nM when PV suffered from natural oxidation (Figure 3A), which was consistent with previously developed V- and DV-based bacterial biosensors toward Pb(II) (Hui et al., 2022b). Considering the antibacterial activity of V, the signal intensities of both non-cytotoxic PV and DV were significantly more robust than that of V under the same concentration of lead exposure (Hui et al., 2022b). As expected, the LOD of the PV-based biosensor is significantly lower than that of fluorescent biosensors resulting from the amplification effect of continuous pigment biosynthesis. Furthermore, no linear relationship was observed between the fluorescent signal and Pb(II) concentration (Wei et al., 2014). A good regression relationship was found in this study. The quantifiable Pb(II) concentration reached 3,000 nM, which was similar to the DV-based lead biosensor but significantly more comprehensive than previously developed cytotoxic V-based biosensors (Hui et al., 2022b), non-cellular biosensors (Zhang et al., 2019; Yu et al., 2022) and cellular fluorescent biosensors (Bereza-Malcolm et al., 2016). In contrast to fluorescent or bioluminescent biosensors, the naked eye could directly distinguish gradient changes of dual-color signals (Figure 3G). The accumulation of visible colorants helps direct the reading of high Pb(II) exposure with the naked eye and the development of visible light colorimetric methods.

The biosensing selectivity largely depends on the metal sensory element. Previous studies showed that group 12 metals exerted some influence on the response of biosensors employing metalloregulator PbrR as a Pb(II) sensory component (Hui et al., 2022b). The developed biosensor comprised a PbrR-based sensory element and a *vioABDE* reporter gene cluster. As expected, excellent biosensing selectivity was demonstrated when PV-based biosensors were exposed to metal ions alone (Figures 4A,B). Only Cd(II) and Hg(II) exerted some adverse effects on the Pb(II) induced signal output (Figures 4C,D). The electrochemical property is similar among Pb, Cd, and Hg (Chen et al., 2005). The Cd(II) and Hg(II) may compete with Pb(II) to occupy the metal binding domains of dimeric PbrR. However, the association with non-target metal cannot efficiently trigger the downstream reporter gene’s transcription (Hui et al., 2022b). The molecular evolution of metalloregulator was always thought to be a promising solution to improve the metal selectivity of biosensors (Jia et al., 2020; Cai et al., 2022).

Soluble inorganic and organic components in natural aquatic systems can change metal morphology, resulting in its differential bioavailability and ecotoxicity (Ryan et al., 2009). It is necessary to validate a novel biosensor in monitoring environmental pollutant metals (Gavrilas et al., 2022). This study developed a simple and three-step detection process to detect residual Pb(II) in the environmental water (Figure 5A). Artificially contaminated environmental water samples were prepared and mixed into the biosensing culture system. Engineered biosensor cells were simulated to be exposed to increased Pb(II) concentrations in four water samples. Although the whole dose–response curves of environmental water are similar to that under experimental conditions, environmental water matrices exerted a specific influence on the generation of pigment signals, particularly on the heterogeneous brown biosensing signal toward Pb(II), which might lead to a differential LOD (Figure 5B) and a narrow quantifiable concentration range (Figure 5F) in detecting various environmental samples. The naked eye quickly identified the gradient changes in color deepening (Figures 5H,I).

Interestingly, a significantly decreased relative standard deviation was observed in the oxidation treatment group compared to the non-oxidation treatment group (Supplementary Tables S3, S4). It can be well explained that PV may be oxidized into homogeneous derivatives under extreme oxidizing conditions. However, the autoxidation of PV in the n-butanol tends to produce more ambiguous products. The above findings suggest that PV-based biosensors, especially employing green signals after oxidation treatment, become a semi-quantitative device powerful in warning high dose Pb(II) pollution through direct reading with the naked eye.

Novel visible pigment-based bacterial biosensors share the same advantages over luminescent and fluorescent bacterial biosensors (Liao et al., 2006; Hui et al., 2021b). It differs from widely used enzyme-based biosensors because no extra substrate is necessary. The pigment-based colorimetric method is also superior to traditional colorimetric methods. The critical devices involved in these biosensors are a cheap incubator, a low-speed centrifuge, and a microplate reader with a visible detection range. Regarding the initial investment in equipment, its cost is far lower than traditional sensors. The biosensing plasmid and host cells are stably stored at low temperatures. Fresh biosensor cells may be transformed regularly to avoid genetic variation. However, the costs associated with bacterial culture, plasmid preservation, and transformation can be ignored. All these properties make metabolically engineered bacteria potentially become low-cost and mini-equipment biosensors (Naik and Dubey, 2013; Guo et al., 2021a).

In conclusion, this study transformed an intermediate of the branched violacein biosynthetic pathway into a dual-color biosensing reporter. The PV-derived dual-color signals had excellent biosensing properties and showed great potential in developing whole-cell biosensors toward pollutants such as toxic Pb(II). Colored signals read directly by the naked eye enabled a PV-based biosensor, a potential on-site detection method. The sensitivity and selectivity of bacterial biosensors are expected to be improved by optimization of genetic circuits and molecular evolution of metalloregulator PbrR in future studies.

## Data availability statement

The original contributions presented in the study are included in the article/Supplementary material, further inquiries can be directed to the corresponding authors.

## Author contributions

C-yH, N-xZ, and HL designed the experimental protocol. D-lZ and C-yH drafted the manuscript. D-lZ, YG, B-cM, M-qL, C-xG, Y-qL, and H-jW carried out most of the study. D-lZ, YG, and B-cM analyzed the data. All authors contributed to the article and approved the submitted version.

## Funding

This work was supported by the National Natural Science Foundation of China (82073517), the Natural Science Foundation of Guangdong Province (2021A1515012472 and 2023A1515011184), the Science and Technology Program of Shenzhen (KCXFZ20201221173602007), Shenzhen Key Medical Discipline Construction Fund (SZXK068), and Shenzhen Fund for Guangdong Provincial High-level Clinical Key Specialties (SZGSP015).

## Conflict of interest

The authors declare that the research was conducted in the absence of any commercial or financial relationships that could be construed as a potential conflict of interest.

## Publisher’s note

All claims expressed in this article are solely those of the authors and do not necessarily represent those of their affiliated organizations, or those of the publisher, the editors and the reviewers. Any product that may be evaluated in this article, or claim that may be made by its manufacturer, is not guaranteed or endorsed by the publisher.
