# Supplementary material for: Pb(II)-inducible proviolacein biosynthesis enables a dual-color biosensor toward environmental lead
Source: Front Microbiol. 2023 Jul 27;14:1218933. doi: 10.3389/fmicb.2023.1218933 (PMC10413148; doi:10.3389/fmicb.2023.1218933)
Supplement: Supplementary file 1 [file Data_Sheet_1.pdf]

Supplementary data

Pb(II)-inducible proviolacein biosynthesis enables a dual-color biosensor toward environmental lead

De-long Zhu[1†], Yan Guo[2†], Bing-chan Ma[3], Yong-qin Lin[2], Hai-jun Wang[2], Chao-xian Gao[2], Ming-qi Liu[1], Nai-xing Zhang[2*], Hao Luo[1*], Chang-ye Hui[1, 2*]

[1] School of Public Health, Guangdong Medical University, Dongguan, China.

[2] Shenzhen Prevention and Treatment Center for Occupational Diseases, Shenzhen, China.

[3] School of Public Health, Tongji Medical College, Huazhong University of Science and Technology, Wuhan, China.

[†]These authors have contributed equally to this work

*Correspondence

Nai-xing Zhang

zhanghealth@126.com

Hao Luo

lh426@gdmu.edu.cn

Chang-ye Hui

hcy_sypu@hotmail.com

Table. S1 The induction coefficient of TOP10/pPb-vioABDE exposed to Pb(II) in the non-oxidation treatment group.

Time (h)	Induction coefficient		Absorbance at 652 nm					
			Background		0.15 μM		1.5 μM	
	0.15 μM	1.5 μM	Mean	SD	Mean	SD	Mean	SD
0	0.976	1.087	0.042	0.006	0.041	0.001	0.046	0.002
1	1.612	1.686	0.040	0.001	0.065	0.009	0.068	0.008
2	1.690	2.214	0.042	0.001	0.071	0.012	0.093	0.020
3	1.500	2.490	0.063	0.028	0.095	0.011	0.158	0.026
4	2.165	3.736	0.077	0.038	0.167	0.032	0.288	0.029
5	2.738	4.527	0.092	0.048	0.251	0.010	0.415	0.033
6	3.179	4.685	0.101	0.045	0.320	0.053	0.472	0.034
7	3.334	5.164	0.096	0.046	0.319	0.063	0.494	0.024

Table. S2 The induction coefficient of TOP10/pPb-vioABDE exposed to Pb(II) in the oxidation treatment group.

Time (h)	Induction coefficient		Absorbance at 652 nm					
			Background		0.15 μM		1.5 μM	
	0.15 μM	1.5 μM	Mean	Standard	Mean	Standard	Mean	Standard
0	0.914	1.108	0.046	0.004	0.042	0.003	0.051	0.013
1	1.984	1.776	0.042	0.003	0.083	0.009	0.074	0.015
2	2.148	2.060	0.050	0.007	0.107	0.032	0.102	0.004
3	2.550	2.921	0.064	0.010	0.162	0.038	0.186	0.018
4	3.725	4.625	0.067	0.008	0.248	0.017	0.308	0.019
5	3.957	6.358	0.077	0.009	0.306	0.011	0.492	0.060
6	3.587	6.047	0.085	0.020	0.304	0.013	0.512	0.052
7	3.131	5.210	0.097	0.026	0.303	0.021	0.504	0.038

Table. S3 The relative standard deviation in the non-oxidation treatment group

Pb(II) nM	Purified water	Tap water	Lake water	Soil extract
0	15.7%	30.7%	7.8%	12.8%
5.86	12.5%	42.6%	15.5%	28.1%
11.7	13.7%	44.8%	19.8%	24.1%
23.4	12.9%	43.1%	14.5%	29.2%
46.9	15.4%	41.2%	14.2%	24.7%
93.8	14.0%	33.7%	14.5%	28.7%
187.5	17.4%	29.5%	13.6%	21.1%
375	17.5%	23.6%	17.0%	19.3%
750	10.6%	15.2%	16.0%	19.2%

Table. S4 The relative standard deviation in the oxidation treatment group

Pb(II) nM	Purified water	Tap water	Lake water	Soil extract
0	18.5%	2.5%	2.2%	5.3%
0.732	2.3%	2.4%	5.2%	6.5%
1.46	2.2%	6.0%	6.4%	7.7%
2.93	2.7%	1.7%	6.3%	5.3%
5.86	2.6%	10.7%	8.0%	8.4%
11.7	7.7%	3.0%	4.5%	9.8%
23.4	7.0%	9.4%	5.0%	8.5%
46.9	4.7%	3.0%	6.8%	8.7%
93.8	8.2%	7.3%	7.2%	2.1%
187.5	2.5%	5.4%	7.9%	8.3%
375	1.9%	7.2%	5.6%	9.3%
750	3.2%	7.6%	3.7%	10.9%

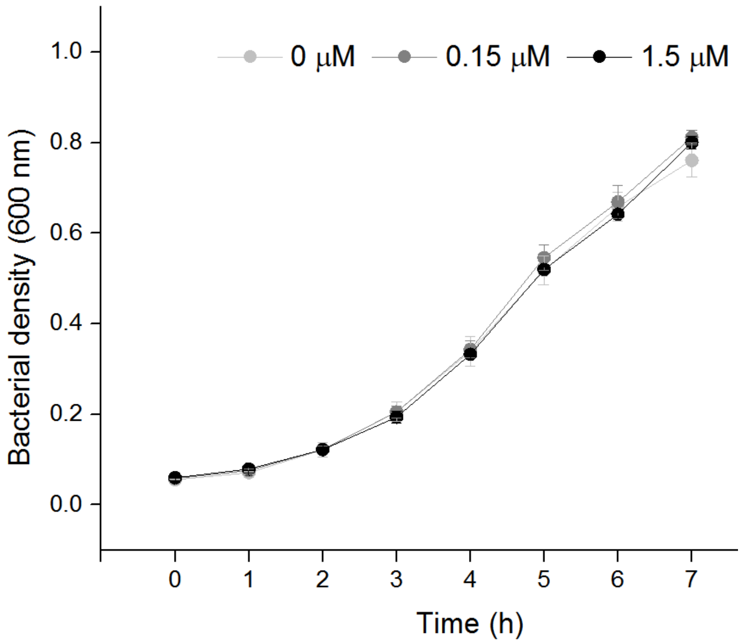

Figure. S1 Bacterial density of TOP10/pPb-vioABDE exposed to different concentrations of Pb(II). TOP10/pPb-vioABDE in the lag phase was induced with Pb(II) at 0, 0.15, and 1.5 μM, and samples were taken at 1 h intervals. Values are mean ± standard deviation (n=3).

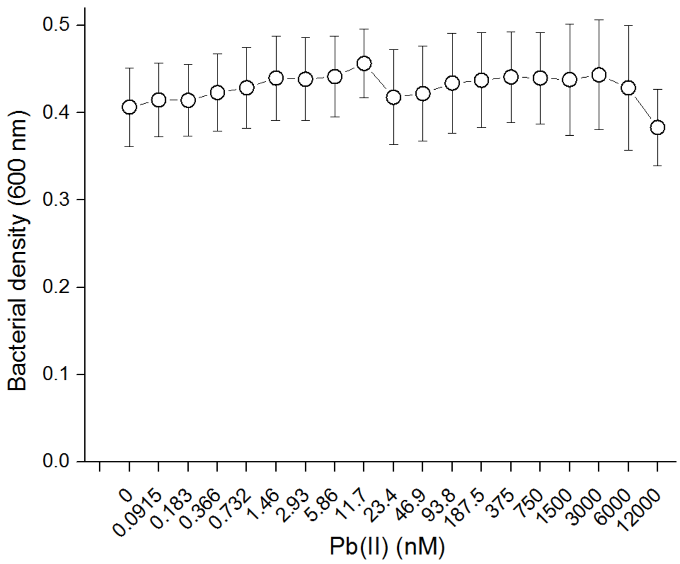

Figure. S2 Bacterial density of biosensors induced with Pb(II) ranging from 0 to 12000 nM. Recombinant TOP10/pPb-vioABDE was induced with increased Pb(II) concentrations, and bacterial density was measured after 5 h induction. Values are mean ± standard deviation (n=3).

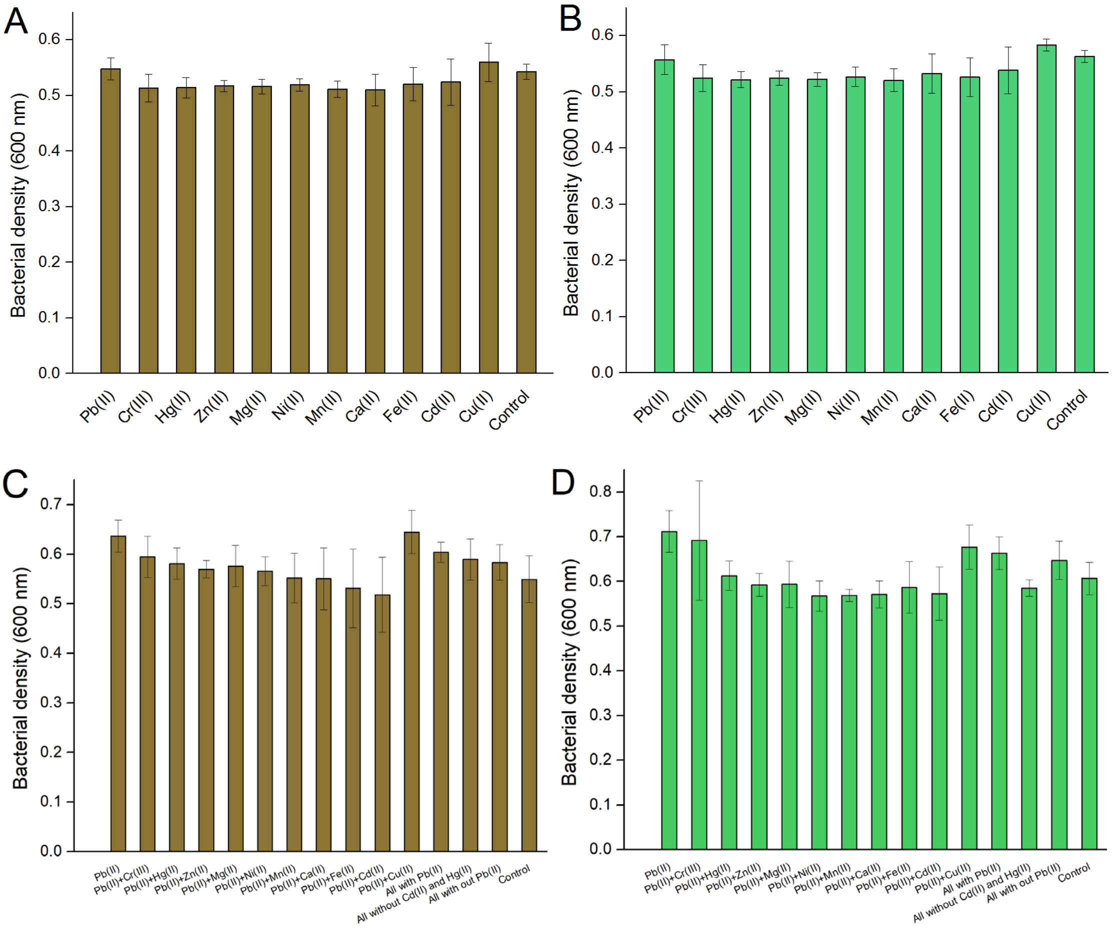

Figure. S3 Bacterial density of biosensors exposed to various metal ions. The bacterial density of TOP10/pPb-vioABDE exposed to various metal ions alone at 1.5 μM in non-oxidation (A) and oxidation (B) treatment groups was determined after culture at 37 °C for 5 h. The bacterial density of TOP10/pPb-vioABDE exposed to various mixed metal ions, all at 1.5 μM in non-oxidation (C) and oxidation (D) treatment groups, was determined after culture at 37 °C for 5 h. Values are mean ± standard deviation (n=3).

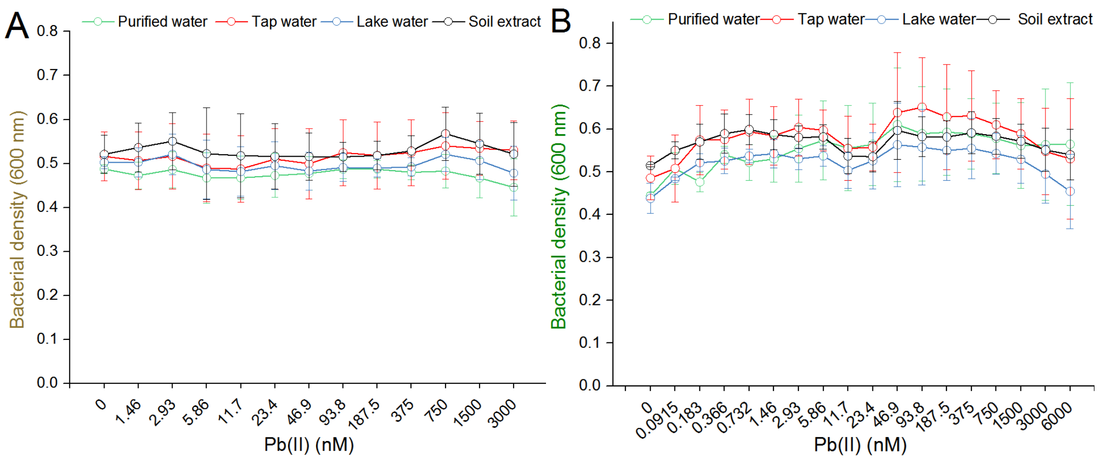

Figure. S4 Bacterial densities of TOP10/pPb-vioABDE after 5-h induction with increased Pb(II) concentrations existing in culture mediums prepared using environmental water samples. (A) In the non-oxidation treatment group, the bacterial density of TOP10/pPb-vioABDE was exposed to 0-3000 nM Pb(II). (B) The bacterial density of TOP10/pPb-vioABDE exposed to 0-6000 nM Pb(II) in the oxidation treatment group. Values are mean ± standard deviation (n=3).

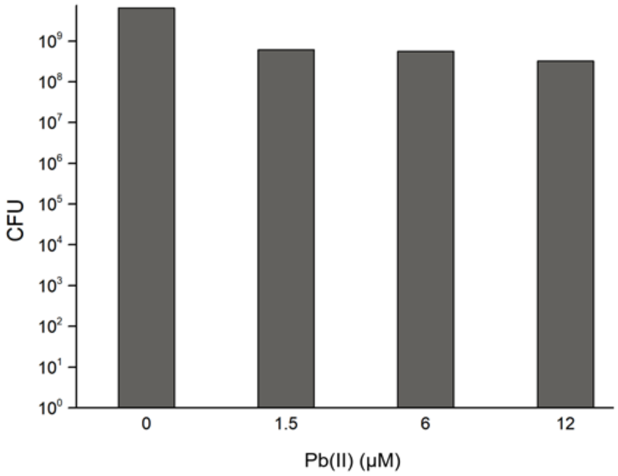

Figure. S5 Colony-forming unit (CFU) of TOP10/pPb-vioABDE after 5-h induction with increased Pb(II) concentrations of 0, 1.5, 6, and 12 μM in LB broth prepared with purified water. Shown is a representative result from three independent assays.
